# Illustrative examples of probable transfer of resistance determinants from food animals to humans: Streptothricins, glycopeptides, and colistin

**DOI:** 10.12688/f1000research.12777.1

**Published:** 2017-10-05

**Authors:** Hattie E. Webb, Frederick J. Angulo, Sophie A. Granier, H. Morgan Scott, Guy H. Loneragan

**Affiliations:** 1 International Center for Food Industry Excellence, Department of Animal and Food Sciences, Texas Tech University, Lubbock, TX, 79409, USA; 2 Division of Global Health Protection, Center for Global Health, Centers for Disease Control and Prevention, Atlanta, GA, 30333, USA; 3 Laboratory for Food Safety, Anses, Université Paris-Est, Maisons-Alfort, F-94701, France; 4 Department of Veterinary Pathobiology, College of Veterinary Medicine and Biomedical Sciences, Texas A&M University, College Station, TX, 77843, USA

**Keywords:** olistin, glycopeptides, streptothricins, antimicrobial resistance, Critically Important Antimicrobials

## Abstract

Use, overuse, and misuse of antimicrobials contributes to selection and dissemination of bacterial resistance determinants that may be transferred to humans and constitute a global public health concern. Because of the continued emergence and expansion of antimicrobial resistance, combined with the lack of novel antimicrobial agents, efforts are underway to preserve the efficacy of current available life-saving antimicrobials in humans. As a result, uses of medically important antimicrobials in food animal production have generated debate and led to calls to reduce both antimicrobial use and the need for use. This manuscript, commissioned by the World Health Organization (WHO) to help inform the development of the WHO guidelines on the use of medically important antimicrobials in food animals, includes three illustrations of antimicrobial use in food animal production that has contributed to the selection—and subsequent transfer—of resistance determinants from food animals to humans. Herein, antimicrobial use and the epidemiology of bacterial resistance are described for streptothricins, glycopeptides, and colistin. Taken together, these historical and current narratives reinforce the need for actions that will preserve the efficacy of antimicrobials.

## Context

Apart from a few molecules, many antimicrobial agents, such as antibiotics, either occur in nature or are derived from natural compounds. Likewise, their corresponding resistance determinants have occurred naturally for millennia. Mounting evidence, however, informs us that decades of global, anthropomorphic antimicrobial overuse has resulted—and is resulting—in the selection and spread of antimicrobial resistant bacteria and their determinants. Much of this antimicrobial use is occurring in food animal production; while some over-selection from this use does not extend to distinctly human pathogens, zoonotic bacteria that can be transmitted from food animals to humans through the food supply and environment may pose an increased risk to humans due to adverse consequences of antimicrobial resistance such as treatment failure. Human deaths attributed to all bacterial resistance are currently estimated to be 700,000 annually ^1^, and—unless action is taken—this estimate is projected by economists to exceed 10 million by 2050, thereby surpassing cancer ^2^.

Many organizations have begun to engage in efforts to reduce the potential public-health impact of bacterial resistance associated with the use of antimicrobials in food animals. In particular, the World Health Organization (WHO) has established an Advisory Group on Integrated Surveillance of Antimicrobial Resistance (AGISAR). This group has been key in producing guidelines on the use of antimicrobials in food-producing animals (hereafter, termed the “guidelines”), the integrated surveillance of antimicrobial resistance, regularly revised lists of critically important antimicrobials for human medicine (CIA List), and supporting capacity building and infrastructure development efforts in the developing world. This review was commissioned in the context of informing the development of the WHO guidelines on use of medically important antimicrobials used in food animals to be published in October, 2017. Therefore, our objective is to provide three specific examples that illustrate selection and subsequent transfer of resistant determinants from food animals to humans. These illustrative examples are streptothricins, glycopeptides, and colistin.

## Limitations

Our general knowledge on antimicrobial resistance among bacteria is ever evolving; in particular, the story of colistin resistance is rapidly unfolding. This review was prepared for the WHO AGISAR meeting of October, 2016 (Raleigh, North Carolina, US) at which time the WHO guidelines was being drafted; therefore, the cited literature is considered up to date as of September 15, 2016. The scope of this review paper was centered on the evidence of antimicrobial use (and amount of use) in food animals and the epidemiology of common resistance mechanisms. Routes of antimicrobial administration were not evaluated or discussed within this report, but likely also play a role in antimicrobial resistance determinant selection. Further, dissemination of bacteria and resistance genes are frequently not unidirectional events. As such, we do not discount the importance of other directional routes of transfer (e.g. direct or indirect transfer from human to animal populations); however, the scope of this review was limited to transfer from food-producing animals to humans. The selection and dissemination of antimicrobial resistance is a complex, multifactorial phenomenon. Unfortunately, there is no ‘perfect’ experiment or controlled environment to demonstrate selection, dissemination, and the subsequent risks imposed through the sharing of resistance determinants among bacterial and host populations, and we acknowledge up front that there remain data gaps.

## Streptothricins

Streptothricins are a distinct group of antibiotic compounds isolated from the genus *Streptomyces* ^3, 4^. The first streptothricin compound (F) was described in 1942 ^5^. Antibiotic agents of the streptothricin group are composed of varying combinations and proportions of the streptothricin compounds (A, B, C, D, E, F, and X) ^6^. More than 70 mixtures of streptothricin compounds have been described and subsequently named, including: streptolin, racemomycin, geomycin, grisein, pleocidin, and nourseothricin; however, the amount of detail available regarding the chemical structure and antibacterial activity of each of the streptothricin antibiotic agents varies greatly. Nonetheless, the streptothricin antibiotic agents are known to be effective against pathogenic fungi and have both bacteriostatic and bactericidal effects on Gram-negative and Gram-positive bacteria through the inhibition of protein synthesis and misreading of genetic information ^7– 9^.

### Usage

Nephrotoxicity associated with streptothricin antibiotic agents has prevented clinical use of these agents in human medicine ^10, 11^. As a result, use of the streptothricin antibiotic agents has been largely limited to plant production and animal husbandry in a select few countries, particularly China and the former German Democratic Republic (GDR; East Germany) ^12, 13^. The most detailed accounts of streptothricin use and the apparent subsequent dissemination of resistance are available from the GDR. Between 1981 and 1989, nourseothricin—a mixture of streptothricin D and F—was used in the GDR for in-feed growth promotion in the swine industry ^5, 14^. No data are available about the amounts of streptothricins or nourseothricin produced, distributed, or used in the swine industry during this time. Nourseothricin was not used in animals in the GDR prior to the introduction of its use in swine, and nourseothricin use in the GDR was limited to the swine industry ^15^. Furthermore, no use of other streptothricin antibiotic agents in animals or humans has ever been reported in the GDR.

### Resistance

It has been reported that, prior to utilization in the GDR swine industry in 1981, acquired nourseothricin resistance in Enterobacteriaceae among animal and human isolates was rare and believed to be solely associated with chromosomal mutations ^12, 16, 17^. Furthermore, when phenotypic resistance was reported, it was never found to be a mobilizable resistance, although the extent of antimicrobial surveillance or screening is not cited and is unknown for that period of time. In 1981, less than one year after the initial use of nourseothricin in the swine industry, a streptothricin-streptomycin-spectinomycin resistance phenotype was observed in *Escherichia coli* isolated from rectal swabs from pigs on multiple farms, “sewage”, and from the feces of those in direct contact with the pigs (i.e. farm personnel) ^17^. This resistance was found to be mediated by streptothricin-acetyltransferase ( *sat*) genes coding for a nourseothricin-inactivating enzyme, which is carried on a transposon, designated Tn1825 ^17^.

### Evidence for transmission

From 1981 to 1983, plasmid-mediated streptothricin resistance was documented in *E. coli* isolated from rectal swabs of pigs being treated with nourseothricin and slurry from their farms in multiple geographical locations within the GDR ^14^. Hummel and colleagues also identified streptothricin-resistant *E. coli* in piglets being treated with nourseothricin, the gut flora of persons with direct contact with the pigs (i.e. farm personnel), the gut flora of persons with in-direct contact with the pigs, who had no other connection to the livestock industry (i.e. farm personnel’s family members), and among the gut flora of outpatients living in the same region that had no apparent contact with pigs ^17, 18^. Remarkably, the authors did not observe streptothricin resistance in samples from piglets or humans in regions where nourseothricin was not being used. Further, the prevalence of streptothricin resistance was highest in *E. coli* isolated from piglets (33% of 306) and declined in the following order: isolates from farm personnel (18% of 377), isolates from farm personnel’s family members (17% of 334), isolates from outpatients in the region (16% of 266) and isolates from urinary tract infections in outpatients in the region (1% of 28).

Despite discontinuation of nourseothricin use in the GDR swine in 1988, the identification of streptothricin resistance and associated resistance determinants continued and broadened. Streptothricin resistance has now been associated with the *sat*, *stat*, and *nat* genes ^19^. In 1992, the first report of streptothricin-resistance *Campylobacter* isolated from pig slurry was published ^20, 21^. Integrons harboring the gene sequence of these resistance determinants have also been observed in other bacteria (clinical isolates, animal environments, and food-producing animals), including *Salmonella enterica*, *Enterococcus faecium*, *Acinetobacter baumannii*, *Burkholderia cenocepacia*, *Vibrio cholerae, Shigella sonnei,* and *S*. *flexneri* ^12, 22– 27^.

Interestingly, the spread of the streptothricin resistance gene to these other ecological niches and bacterial populations has occurred without direct selection pressure (i.e. use of streptothricins in animals or human medicine) ^12^. Importantly, the streptothricin resistance genes are often harbored in integrons with resistance determinants present to other antimicrobial agents, namely determinants coding for resistance to streptomycin, spectinomycin, trimethoprim, or kanamycin ^22– 25^. It is possible that such co-resistance may have contributed to the early dissemination of streptothricin resistance, but the early epidemiological studies did not report information on use of other antimicrobial agents. Little to no information is provided about the animals and humans from which the isolates were collected. Furthermore, because there were few studies that searched for streptothricin resistance prior to the 1980s, it is not known if streptothricin resistance determinants were present in bacteria before this time. Nonetheless, this illustrative example outlines the published account of the likely emergence and dissemination of plasmid-borne resistance from swine to humans.

### Summary

Nourseothricin, a streptothricin antimicrobial agent, was widely used as a growth promoter in the swine industry in the former German Democratic Republic from 1981–1988. In contrast, toxicity prevented use of streptothricin antimicrobial agents in humans. Less than one year after the introduction of nourseothricin in swine, a plasmid-borne streptothricin resistance ( *sat*) seemingly emerged in *E. coli* isolated from swine administered nourseothricin. Subsequently, plasmid-borne streptothricin resistance was detected in the gut flora of humans with direct, indirect, and no contact to pig farms, but living in the same regions. Following reports of the plasmid-mediated streptothricin resistance demonstrates an illustrative example of the detection—and apparent emergence—of streptothricin-resistant bacteria in swine as a result of antimicrobial use, and the dissemination of the resistant bacteria and mobile genetic elements conferring resistance to humans.

## Glycopeptides

Glycopeptides are a broad-spectrum antimicrobial class, including vancomycin, and its derivatives teicoplanin, telavancin, dalbavancin, oritavancin, and avoparcin ^28^. Glycopeptides block cell wall assembly in Gram-positive bacteria by inhibiting peptidoglycan synthesis ^28^. Therefore, the clinical importance of the glycopeptide class has been the treatment of infections caused by Gram-positive pathogens. For a large part of the 1980s and 1990s glycopeptides were the drugs of last-resort for multidrug-resistant Gram-positive infections in humans ^29^.

### Usage

Vancomycin, the first antibiotic of the glycopeptide class, was first described in 1955 and was subsequently approved for human use by the United States (US) Food and Drug Administration (FDA) in 1958 ^29– 31^. The dates of approval and beginnings of human use in European countries are unknown. Renal toxicity and ototoxicity (largely due to impurities in the drug) limited vancomycin use in humans until the early 1980s when multidrug-resistant Gram-positive bacteria began to emerge and purified formulations of vancomycin became available ^32, 33^. Annual vancomycin usage in humans in the US climbed from 2,000 kg in 1984 to 11,460 kg in 1994 ^33^. In Europe and Australia, human vancomycin use was more limited ^33^; for example, in Australia, an average of 193 kg of vancomycin was used in humans annually between 1991 and 1993 ^34, 35^. France reported 200 kg of vancomycin was used in humans in 1984, increasing to only 1,151 kg in 1994 ^33^. Annual vancomycin usage in humans in Germany, Italy, United Kingdom (UK), the Netherlands, and Denmark each ranged between 24 to 408 kg in 1994 ^33, 36^. Human use of vancomycin began to decline after 1994 following efforts to promote vancomycin conservation, an attempted to limit dissemination of glycopeptide-resistant bacteria.

Although vancomycin use in humans in Europe was very limited in the 1990s, avoparcin, a glycopeptide antimicrobial, was heavily used in many European countries and Australia as an antimicrobial growth promoter in livestock ^34^. Avoparcin use for growth promotion is documented in Europe as early as 1975 and products containing avoparcin have been registered in Australia since 1978 ^37– 39^; while data supporting heavy use of avoparcin in many European countries are limited, data from Denmark indicate 24,000 kg of active avoparcin were used in swine and broilers in 1994 ^36^. Austria reported an average of 62,642 kg of avoparcin for animal production use were imported per year from 1992 to 1996 ^40^. Australia used an annual average of 125,000 kg of avoparcin between 1991 and 1993 ^34, 35^. Avoparcin has never been licensed for use in animals in the US ^41^. Following the isolation of glycopeptide-resistant bacteria from food animal products at the retail level, attempts to mitigate the risk of human exposure to glycopeptide-resistant enterococci (GRE) through the food chain led to the ban of avoparcin for growth promotion use in Denmark and Norway in 1995, Germany in 1996, followed by the remaining European Union member states in 1997, and withdraw of avoparcin from the Australian market in 2000 ^20, 38, 39, 42– 46^.

### Resistance

Transferable glycopeptide resistance in enterococci was first reported in human patients in both France and the UK in 1986, and then in the US in 1987 ^47– 49^. However, it wasn’t until the 1990s that considerable attention turned to the evaluation of glycopeptide use and resistance due to differing epidemiological trends between GRE in the US and Europe. In the US in the 1990s, GRE emerged as a significant cause of healthcare-associated infection and colonization in many hospitals—frequently associated with the high use of vancomycin in those hospitals ^50– 52^. Hospital-associated GRE infections rose at an endemic rate; with the proportion of vancomycin resistant enterococcal blood isolates climbing from little to no resistance in 1989 to 25.9% in 2000 ^39, 53^. In the 1990s in Europe, prevalence rates of GRE in hospitals remained low; however there were reports of GRE in healthy human carriers in the community (e.g. people with no association to a hospital) and sporadic hospital outbreaks ^54– 56^.

Monitoring of antimicrobial resistance to growth promoters was not common practice prior to the mid-1990s ^57^. Perhaps as a result, the first detection of GRE isolated from sewage, animals, and healthy humans in the community (i.e. outside of hospitals) were reported in the mid-1990s ^39, 42, 45, 56, 58– 66^. Notably, an association was made between use of avoparcin and the occurrence of GRE in livestock and their environments in Belgium, Denmark, Finland, France, Germany, UK, and the Netherlands—directing a spotlight to food animal production ^42, 50, 51, 57, 60, 61, 65, 67– 74^.

The differing epidemiological trends in GRE between the US and Europe led to considerable interest to compare GRE from European farm animals fed avoparcin, hospitalized humans, and non-human sources using various molecular methods ^32^. Such investigations provided a great deal of insight about the epidemiology of acquired resistance genotypes associated with glycopeptide resistance, particularly the most globally widespread and prevalent glycopeptide resistance in enterococci, *vanA* resistance. *vanA* is an inducible resistance to vancomycin and often teicoplanin mediated by a complex cluster of resistance genes ( *ORF1, ORF2, vanR, vanS, vanH, vanA, vanX, vanY,* and *vanZ*) often carried on a 10,851 bp transposon designated Tn *1546* ^67, 75– 77^.

### Evidence for transmission

Analysis of GRE with *vanA* resistance revealed a certain level of host-association ^51, 78– 80^. Reports using deoxyribonucleic acid (DNA) sequence typing and phylogenetic analysis for genotyping clustered *vanA Enterococcus faecium* isolates from varying ecological backgrounds into distinct genogroups. Strains collected from pigs and healthy people often clustered together forming a single genotype or cluster. In contrast, isolates collected from poultry and their farmers, veal calves and their farmers *,* and hospitalized patients from epidemics worldwide each form genetically distinct clusters ^78– 80^. One of the first insights of genetic relatedness was the observation of a single base change (G8234T) in the *vanX* of Tn *1546*, which was first described by Jensen *et al.* ^50, 51^ The G-variant was associated with isolates collected from poultry and poultry farmers in multiple countries ^51, 57, 68, 81^. The T-variant, on the other hand, was predominantly observed in swine isolates from differing countries ^51, 80^. Interestingly, both G- and T-variants were associated with isolates likely of human origin ^51^. In fact, it was observed that all human samples from a Muslim country—a population that likely eats little or no pork—belong to the G-variant associated with poultry, thus further suggesting GRE transmission may occur between food animals and humans ^51^.

Further investigation of *vanA* mechanism by Willems *et al.* ^80^, revealed amplified fragment length polymorphism (ALFP) genotyping clustered a bank of 255 *E. faecium* isolates from various ecological niches and geographic locations into four genogroups (designated A–D). All isolates collected from pigs and 76% of isolates collected from healthy people clustered to form Genogroup A. Almost all isolates collected from poultry (95%) and 50% of isolates from poultry farmers clustered to form Genogroup B, and Genogroup D contained 70% of isolates collected from veal calves and their farmers *.* Further, 84% of isolates collected from hospitalized patients from epidemics in the UK, US, and Australia formed a genetically distinct cluster from the healthy humans and animal genogroups, which the authors designated Genogroup C ^80^. Similar findings have been demonstrated using various other genotypic methods ^39, 72, 78, 79, 82– 84^.

The VanA gene cluster is now one of many described genotypic determinants encoding glycopeptide resistance, and the early genotypic studies described herein only evidence the likely dissemination of a single glycopeptide resistance determinant from animals to healthy people. Further, the differing epidemiological trends between the US and Europe detail two situations that consequently led to the selection of glycopeptide resistance determinants in distinct ecological niches—one in hospitalized patients and the other in healthy humans and animals. Nonetheless, the genetic characterization of the VanA gene cluster provides an illustrative example of the dissemination of glycopeptide resistance from animals to humans following selection, due to use of avoparcin for growth promotion.

### Summary

Avoparcin appears to have been widely used in food animals, particularly in chickens and pigs, in parts of Europe, since before the mid 1970s. Vancomycin use in humans, in contrast, was very limited in Europe until the late 1990s. It appears likely that the use of avoparcin in food animals selected for the emergence and dissemination of a resistance gene cluster (VanA), which was increasingly identified in animals and healthy people. Molecular subtyping of the VanA gene cluster has identified variants that are more likely to be associated with certain food animal species. Subsequently, GRE were transmitted and found to colonize healthy humans, presumably via the food chain. Therefore, evaluation of the VanA gene cluster variants provides an illustrative example of the emergence and selection of a genetic resistance determinant as a consequence of antimicrobial use in food animals, and subsequent dissemination of the resistant bacteria to humans.

## Colistin

Polymyxin E (herein simply referred to as colistin) is a cationic, multicomponent lipopeptide antimicrobial agent of the polymyxin family that was first discovered in 1949 and isolated in 1950 ^85^. Polymyxins are effective against Gram-negative bacilli through their affinity to bind to the negatively charged lipopolysaccharide (LPS) of the cell outer membrane ^86^. This binding, more specifically to the anionic lipid A of the LPS, leads to disruption of the cell membrane integrity, ultimately leading to leakage induced cell death ^86– 88^. Two forms of the colistin compound are available for clinical use: colistin sulfate (colistin S) and the pro-drug, colistimethate sodium (colistin methanesulfonate sodium, colistin sulfomethate sodium, colistin M).

### Usage

The US FDA first approved colistin for human use in 1962—in the form of colistin sulfate; this first approval was for ear drops ^89^. The FDA subsequently approved a product for injection—in the form of colistimethate sodium—for human use in 1970 ^90^. No US data are available on the quantities of colistin used in humans, although use in the US is thought to have been very low as parenteral use in human medicine quickly fell out of favor due to initial reports of nephro- and neurotoxicity ^90– 96^. More recently, colistin has reemerged as an antimicrobial of interest as a last-resort treatment option for life threatening human infections of multidrug-resistant Gram-negative bacteria, particularly *Pseudomonas aeruginosa, Acinetobacter baumannii* strains, and carbapenem-resistant Enterobacteriaceae ^97– 102^. Approval dates for human use of colistin products in member states of the EU are not clear; however, it is believed that human use began in the 1960s. More recent estimates of polymyxin consumption in humans are available in the EU/European Economic Area ^103^. A sum of 0.8 tonnes of active polymyxin ingredients—including colistin and polymyxin B—were consumed by humans in 22 European countries in 2012 ^104^. In 2014, polymyxin consumption in humans in Europe was 0.012 defined daily doses (DDD) per 1,000 inhabitants—a 50% increase since the 0.008 DDD per 1,000 inhabitants was reported in 2010 ^105^. Countries reporting highest use of polymyxin in humans include Greece, Italy, and Slovakia (0.095, 0.025, and 0.025 defined daily doses per 1,000 inhabitants, respectively) ^105^.

In animals, the extent of colistin sales and use is largely unknown outside of the EU ^106– 109^. In the US, one colistin product, in the form of an injectable colistimethate sodium, was approved for use in chickens in 1998 ^110^; however its marketing status is unclear. In Canada, colistin is not approved for veterinary medicine; however, a loophole in regulation leaves opportunity for “own-use importation,” meaning farmers may import—and use—unlicensed, non-prescription antimicrobials in their animals ^111^. As such, use in swine production has been explored under the veterinarian’s liability (dose, withdrawal period) ^112, 113^. In the EU, colistin-containing products for use in animals are authorized ^114^, though marketing authorization is on a national level and little historical information is available. It is believed that colistin has been used in food animals in the EU since the 1950s ^103^. Colistin is chiefly administered as an oral group treatment in food-producing species to alleviate and prevent Gram-negative infections of the gastrointestinal tract ^107^. Such use is predominantly reported in pigs, poultry, cattle, sheep, goats, and rabbits; however, colistin is also used in laying hens and milk-producing cattle, sheep, and goats ^106, 107^. To date, no data are available that would allow comparison among uses in differing animal species on a European level.

Colistin is also reported to be used in food animal production in Asia, although publically available data are scarce. In China, approximately 90% of the 17.5 million tonnes of colistin produced in 2014 were reportedly consumed by the domestic agriculture industry ^108^. If so, China likely represents the largest colistin producer and consumer in the world. In comparison, a sum of 545.2 tonnes of active polymyxin ingredients—including colistin and polymyxin B—were consumed by food-producing animals, primarily in poultry and swine, in 22 European countries in 2012 ^104^. In 2013, polymyxins were estimated to be the fifth most commonly sold antimicrobial class (7%) for food-producing animals across the EU ^107^. Reported consumption of colistin in animals varied greatly, ranging from <0.2 tonnes in Slovenia, Sweden, Ireland, and Luxembourg to >100 tonnes in Germany, Italy, and Spain ^104^. In another report, annual colistin use in animals in Europe ranged between 0 mg (Finland, Iceland, and Norway) to more than 20 mg (Italy and Spain) per kg of animal biomass ^115^.

Use of colistin for growth promotion in China was banned effective November 1, 2016—which was expected to decrease colistin use in food animal production in China by an estimated 8,000 tonnes ^116^. In March 2015, the European Commission adopted a Decision restricting indications, target species, duration of treatment, and added prudent use warnings to products administered orally to animals that contain colistin as the sole active ingredient ^117^. Evidently, such conversations have continued, as the European Commission recently implemented a Directive to withdraw marketing authorizations for all veterinary medicinal products containing colistin in combination with other antimicrobial substances to be administered orally ^118^. The European Medicines Agency issued a recommendation advising colistin to be used solely as a second line treatment in animals and for sales to be minimized EU-wide ^103^. In Canada, the “own-use importation” loophole has been acknowledged and regulation changes have been proposed that would prohibit such practices ^119^.

### Resistance

Despite widespread and continuous veterinary use, data gaps persist around colistin resistance. Lack of agreement on standardized *in vitro* screening methods and interpretation criteria has complicated and hindered phenotypic surveillance efforts ^86, 120– 123^. This dilemma is largely a consequence of two important colistin characteristics: a large molecule size—which reduces its rate of diffusion into media—and its affinity to adhere to plastics—which are commonly used in phenotypic methods ^86, 123^. Until recently, colistin resistance was believed to be extremely rare; however, surveillance efforts were minimal. In fact, mandatory EU monitoring for colistin resistance in *Salmonella* and *E. coli* only began in 2014 ^124, 125^. Even so, many member states have reported technical difficulties in using the only recommended screening method (i.e., broth dilution) ^103^.

Before November 2015, described phenotypic colistin resistance was associated with chromosomal mutations, which, at least in theory, would be limited to vertical (clonal) dissemination ^86, 126^. However, this previous belief was proven too narrow by the description of a novel, conjugable plasmid-mediated gene conferring colistin resistance ^108^. The gene, designated mobile colistin resistance, or *mcr-1*, was described in *E. coli* and *Klebsiella pneumoniae* isolated from human clinical isolates, retail meat, and food animals in China, between 2011 and 2014 ^108^. The discovery prompted an immediate worldwide response with screening via genomic data mining exercises or else a combination of phenotypic and polymerase chain reaction (PCR)-based methods ^127– 133^. It has now been retrospectively identified with 100% homology in other members of the Enterobacteriaceae family isolated from human, animal, food, and environmental samples and from multiple continents ^133– 140^.

### Evidence for transmission

In humans, the earliest identified *mcr-1* was found in a *Shigella sonnei* isolate arising from a hospitalized child with diarrhea in Vietnam in 2008 ^141^. Bacteria harboring *mcr-1* have also been reported in isolates from humans (both infected patients and asymptomatic human carriers) in Canada ^142, 143^, China ^108, 136, 144– 154^, Denmark ^106, 140^, Ecuador ^155^, Egypt ^156^, France ^130, 157^, Germany ^137, 158, 159^, Hong Kong ^159, 160^, India ^161, 162^, Italy ^159, 163– 165^, Laos ^130^, Malaysia ^159, 166^, Netherlands ^131, 167– 170^, Norway ^171^, Poland ^159, 172^, Portugal ^173^, Russia ^159^, Saudi Arabia ^174^, Singapore ^175, 176^, South Africa ^177, 178^, Spain ^159, 179^, Sweden ^180, 181^, Switzerland ^182– 185^, Taiwan ^186^, Thailand ^130, 187^, United Arab Emirates ^174^, UK ^188^, US ^159, 189– 191^, Venezuela ^192^, and Vietnam ^141, 193^. Bacteria harboring the *mcr-1* gene sequence have likewise been documented from food samples on multiple continents ^108, 129, 136, 138, 140, 142, 168, 173, 185, 186, 188, 194– 199^, suggesting this may be an important route of dissemination from animals to humans.

To date, the earliest identified *mcr-1-*positive isolates are three *E. coli* isolates collected from chickens in China during the 1980s ^200^. Interestingly, *mcr-1* has not been detected in isolates arising during the two subsequent decades; however, the reported proportion of *mcr-1*-positive isolates in China begins increasing in 2009 ^200^. Furthermore, in Europe the earliest *mcr-1-*positive isolate was identified as an *E. coli* originating from a diarrheic veal calf in France in 2005 ^132^. Observations of *mcr-1* in bacteria isolated from food-producing animals, their products, or environments now includes: pigs (Belgium ^128, 201^, Brazil ^202^, China ^203, 204^, France ^127^, Germany ^137, 205, 206^, Japan ^133, 207^, Laos ^130^, Malaysia ^136, 138, 166^, Spain ^208^, Taiwan ^186^, Venezuela ^192^, Vietnam ^209, 210^, UK ^211^, US ^212^), poultry (Algeria ^130, 213^, Brazil ^202, 214, 215^, China ^216– 218^, Denmark ^199^, Egypt ^218^, France ^127^, Germany ^205^, Italy ^106, 219^, Malaysia ^136, 138, 166^, Netherlands ^169^, South Africa ^220, 221^, Spain ^208^, Taiwan ^186^, Tunisia ^185^, Vietnam ^193, 209^), and cattle (Belgium ^128^, Denmark ^199^, Egypt ^222^, France ^127, 132, 223^, Germany ^205^, Japan ^133^, Netherlands ^169^).

Widespread reports of *mcr-1* shortly after its initial characterization indicate the gene was likely being disseminated in an uncharacterized state, and thereby undetected rather than not being present, for a long period of time. The gene has evidently been widely disseminated geographically, as well as across multiple bacterial species of differing origins. Thus far, *mcr-1* has mostly been reported in *E. coli*, although *mcr-1*-positive *Citrobacter* ^224, 225^, *Klebsiella* ^108, 157, 175, 224^, *Shigella* ^141^, *Enterobacter* ^144, 160, 176, 224^, and *Salmonella* ^129, 135, 136, 173, 188, 195, 208, 211, 217^ spp. have also been documented. Furthermore, *mcr-1* has been observed in bacteria from wild animals and water samples, indicating the resistance determinant has also disseminated into the environment ^194, 224, 226– 230^.

Retrospective screening for colistin-resistant bacteria may be limited by the availability of historical isolates and their genomic data. Further, lack of standardized phenotypic screening methods and the delay in genotypic description have likely lead to the underestimation of colistin resistance; nonetheless, the identification and description of the gene has opened the door for screening via genotypic methods. Nevertheless, resistance is still believed to be rare, particularly in humans and in some regions of the world. The initial paper reported the *mcr-1* gene sequence in 1.4% of 902 *E. coli* and 0.7% of 420 *Klebsiella pneumoniae* clinical isolates in China; however, prevalence among *E. coli* isolates originating from pigs and retail meats in China were surprisingly higher: 20.6% of 804 isolates from pigs at slaughter collected between 2012–14 and 14.9% of 523 isolates from retail meats (chicken and pork) collected between 2011–2014 ^108^. Still, in the US the *mcr-1* gene sequence is rare. It was detected in one *E. coli* isolate out of 949 animal intestine samples screened and was not detected in more than 44,000 *Salmonella* and 9,000 *E. coli* and *Shigella* isolates from the National Antimicrobial Resistance Monitoring System (NARMS) and National Center for Biotechnology Information (NCBI) genomic database ^212^. In many reports to date, phenotypic screening is frequently performed prior to genotypic screening. For example, in France, Perrin-Guyomard and colleagues report *mcr-1* in 0.3% of 590 isolates from healthy pigs in 2011–13, 1.8% of 227 isolates from broilers in 2014, and 5.9% of 239 isolates from turkeys in 2014 ^127^; importantly, screening for *mcr-1* was performed only on isolates with a colistin minimum inhibitory concentration > 2 mg/L. Some limitations are inevitable with this approach, as it implies a level of dependency on the much-debated breakpoints and phenotypic methods.

The technological response afforded by genomics-based methods is also not without limitations, especially by not detecting variants of *mcr-1*. In fact, on July 7, 2016, the first account of *mcr-2*, a seemingly distinct gene also conferring colistin resistance was described in *E. coli* isolated from calves and piglets in Belgium ^231^. This *mcr-2* appeared to be more prevalent than *mcr-1* among colistin-resistant *E. coli* of porcine origin ^231^. Then, on July 11, 2016, the first functional variant of *mcr-1*, designated *mcr-1.2*, was reported in *K. pneumoniae* isolated from a surveillance rectal swab of a child in Italy ^232^. Since this report was prepared, a number of other *mcr* variants have been reported, some of which also appear to have been disseminating globally prior to characterization ^233– 235^. While some of these genes may also contribute towards evidencing selection—and subsequent dissemination—of the colistin resistance determinant from food animals to humans, the focus of this report was the initial epidemiology of colistin resistance (i.e. *mcr-1*). Very likely, there remain additional yet-to-be-characterized mechanisms of colistin resistance. Much more work is needed to explore other mechanisms of resistance and to fully comprehend the overall prevalence of colistin resistance determinants and their phenotypic characteristics.

### Summary

Colistin has been widely used in food animals—particularly poultry and swine—in areas of Europe and Asia for decades, perhaps since the early 1980s or earlier. In contrast, colistin use in humans has been extremely limited, at least until recently. It appears highly probable that the use of colistin in food animals has selected for a novel resistance gene ( *mcr-1*), identified as far back as the mid-1980s in chickens in China, which has become increasingly identified in isolates from food animals in many regions of the world since its discovery in 2015. This novel resistance gene has more recently been identified among isolates from humans; however, to date *mcr-1* has been more frequently associated with food animal and meat isolates compared to human isolates. Prevalence of *mcr-1* in animal samples—and to some degree in human samples—appears to be proportional to its use in animals. These chains of events, despite the data gaps, provide an illustrative example of the emergence, selection, and widespread dissemination of a resistance gene as a consequence of antimicrobial use in food animals, and subsequent transfer of bacteria harboring that resistant gene to humans.

## Conclusions

In this review, we have focused on three illustrative examples (i.e. streptothricins, glycopeptides, and colistin) of selection—and subsequent transfer of antimicrobial resistance determinants from food animals to humans. The use of antimicrobials in food animal production contributes to the selection and dissemination of antimicrobial resistance determinants that may reach human populations. However, this review is only part of the picture if taken in a One Health perspective. Its objectives do not encompass the impact of other industries (i.e. environment, human, companion animals, etc.) that also contribute to selection of antimicrobial resistance and it’s consequences on each health sector. To tackle the problem of selection and dissemination of antimicrobial resistance in a true One Health perspective, there is need to fully investigate the role of each of those industries. Nevertheless, the three examples we have described serve to illustrate that use of antimicrobials in food animals can result in antimicrobial resistance that can be transmitted to humans. Therefore, these illustrative examples support the need for actions, such as the proposed WHO Guidelines on use of medically important antimicrobials in food animals, to mitigate the risk of adverse human health consequences resulting from the use of antimicrobial agents in food animals.
