# Supplementary material for: Illustrative examples of probable transfer of resistance determinants from food animals to humans: Streptothricins, glycopeptides, and colistin
Source: F1000Res. 2017 Oct 5;6:1805. [Version 1] doi: 10.12688/f1000research.12777.1 (PMC5686510; doi:10.12688/f1000research.12777.1)
Supplement: Peer review history

# Peer review: Referee response for version 1

**DOI:** 10.5256/f1000research.13843.r27472

**Published:** 2017-11-14

**Authors:** Séamus Fanning

**Affiliations:** 1 UCD-Centre for Food Safety, Science Centre South, University College Dublin, Dublin, Ireland

In this review, Illustrative examples of probable transfer of resistance determinants from food animals to humans: strepothricins, glycopeptides and coilstin, these authors provide a timely update on this important topic of interest to veterinary public health. The three selected antimicrobial agents highlighted are described so as to provide the reader with a background on their uses; the emergence of resistance to these compounds and possible routes of dissemination. Two of the three examples provided (streptothricin- and glycopeptide-resistance) are historical in nature, whilst the colistin-resistant chapter is more recent. The selection of these topics clearly demonstrated the consequences of antimicrobial compound usage and the subsequent response from bacteria of importance to animal- and human-health.

 The manuscript is well designed and clearly written, as presented. It is scientifically sound. The topic is of immediate interest and would serve as a very useful and comprehensive review of our current understanding of this subject. In places the authors highlight some of the limitations in current approaches to the study of some of these resistance mechanisms, particularly in regard to colistin.

I have read this submission. I believe that I have an appropriate level of expertise to confirm that it is of an acceptable scientific standard.

# Peer review: Referee response for version 1

**DOI:** 10.5256/f1000research.13843.r27473

**Published:** 2017-11-03

**Authors:** Wolfgang Witte

**Affiliations:** 1 Robert Koch-Institut (RKI), Wernigerode, Germany

Manuscript “Illustrative examples of probable transfer of resistance determinants from food animals to humans: Streptothricins, glycopeptides, and colistin” describes evidence for antibiotic resistance gene transfer from bacteria colonizing / infecting livestock to bacteria colonizing / infecting humans by means of three examples: streptothricine-resistance and glycopeptide resistance as historical examples, and *mcr* mediated resistance to colistin as an important recent example. Altogether this review is well written and based on a very careful literature research together with a balanced selection of the really relevant publications. Above all the comprehensive and condensed presentation of studies on colistin resistance is impressing.

 A few points will need attention:

- A short discussion on the consequences of the ban of avoparcin as growth promoter would be of interest (e.g. significant reduction of gastrointestinal colonization of healthy humans in the community, Klare et al., 1999). It should also be mentioned that despite this ban several European countries faced an increase of VRE among isolates from blood cultures (EARSnet) since 2004. The question whether the ban was of little significance for the VRE situation in human medicine or whether it could have come much more worse in case of a continuing existing van gene pool in the community cannot be answered in retrospect.
- For the chapter on colistin resistance I suggest to mention that the extent to which *mcr* contributes to colistin resistance in clinical isolates cannot exactly be assessed so far, and that there are cases of acquisition of *mcr* by carbapenemase producing *K.pneumoniae *(Newton-Food et al., 2017). Furthermore, the emergence of *E.coli* harbouring *mcr*-1 and *bla*_KPC-2 _after meropenem and colistin therapy is of interest (Tacao et al., 2017).

I have read this submission. I believe that I have an appropriate level of expertise to confirm that it is of an acceptable scientific standard.
